# Knowledge map of transcranial magnetic stimulation for insomnia: a bibliometric and visual analysis

**DOI:** 10.3389/fpsyt.2025.1682275

**Published:** 2026-01-12

**Authors:** Yanqing Zhao, Xuefei Wang, Li Huang, Wentao Li

**Affiliations:** Shanghai Municipal Hospital of Traditional Chinese Medicine, Shanghai University of Traditional Chinese Medicine, Shanghai, China

**Keywords:** transcranial magnetic stimulation, insomnia, VOSviewer, CiteSpace, bibliometric analysis

## Abstract

**Background:**

Insomnia is one of the most prevalent sleep disorders, severely impacting an individual’s physical and mental wellbeing, diminishing work efficiency and alertness levels, and potentially even causing accidents, thereby exacerbating the economic burden on both individuals and society. The primary treatments for insomnia encompass pharmacological therapy and non-pharmacological therapy. Given the potential side effects associated with pharmacological therapy, there is an urgent need for the application of safe and effective non-pharmacological interventions. Technologies like repetitive transcranial magnetic stimulation (rTMS) have gained widespread adoption in clinical practice globally due to their non-invasive nature, penetration capabilities, and ease of operation.

**Objective:**

The study aimed to probe the trend direction and map the knowledge domain of TMS for insomnia through bibliometrics.

**Methods:**

Publications cognate to TMS for insomnia were retrospectively collected from the WoS database, PubMed, and Scopus from 1980 to 31 December 2024. Next, these data were compiled into scientific maps using the VOSviewer and CiteSpace software.

**Results:**

The cumulative publication trend is increasing yearly, and the growth becomes more apparent since 2016. Moreover, the countries with the highest yield are the USA and China. Collaboration between institutions is more focused on universities in the USA and China. Wang Y is the most prolific author, the “Sleep” journal has the most publications, and the most meaningful journal is “Neuroimage.” Journals tend to lay particular stress on neuroscience majors. Riemann D is the most cited author, and depression, anxiety, and double-blind are the high-frequency research topics in this field. Huang ZY’s 2018 paper published in the Brain Stimulation journal is an important reference. Randomized trials of TMS for insomnia and systematic reviews are the main contents.

**Conclusion:**

Through scientometric analysis of studies on TMS for insomnia, we visualize the involvement of countries, authors, institutions, cited authors, keywords, and cited references using a knowledge graph. Cluster analysis has revealed the primary research areas in this domain, focusing on the preference for low-frequency TMS in treating insomnia and the superior therapeutic outcomes achieved through stimulating the dorsolateral prefrontal cortex. These discoveries serve as a guide for clinical practitioners in conducting subsequent research endeavors. This is crucial for swiftly and precisely identifying key information in this field.

## 1 Introduction

Insomnia is one of the most universal sufferings in clinical practice, characterized by difficulty falling asleep and maintaining sleep, or non-restorative sleep, accompanied by obvious daytime symptoms such as fatigue, decreased attention, impaired cognitive function, irritability, anxiety, and low mood (1). It is estimated that approximately 30% of people worldwide experience one or more insomnia symptoms (2). Insomnia not only increases the risk factors for cardiovascular disease, diabetes, obesity, neurobehavioral dysfunction, and other related diseases but also increases the risk of death in patients (3, 4). The curative treatment for insomnia predominantly embodies drug therapy and non-drug therapy. Although medication can improve sleep in a relatively short period, approximately two-thirds of patients still exhibit a chronic course with fluctuating symptoms (5). Non-pharmacological therapy mainly includes cognitive-behavioral therapy and physical therapy. Cognitive-behavioral therapy is an effective method without worrying about adverse drug reactions, but it is difficult to implement in clinical practice and often has unsatisfactory results. Physical therapy mainly includes rTMS, phototherapy, and biofeedback therapy. Compared with the above treatments, physical therapy has the advantages of small side effects, basic non-invasiveness, stronger operability, and being more economical, and the patient’s acceptance and cooperation are also higher (6). Among them, rTMS technology is increasingly attracting the attention of insomnia patients due to its ability to improve sleep quality, ameliorate sleep structure, and persist therapeutic effects (7). Research on its application in insomnia treatment is steadily gaining momentum (8–11). Recently, rTMS has emerged as a potential therapy for insomnia by modulating neuronal activity and neural plasticity. Specifically, low-frequency rTMS operates by inhibiting cortical excitability and decreasing elevated metabolic levels in insomnia patients (12). Furthermore, studies are also exploring its mechanism of action in regulating synaptic plasticity and functional connectivity to ameliorate impaired brain function or dysfunctional networks (13).

rTMS not only serves as a treatment for insomnia but also offers general benefits in addressing sleep disorders. For instance, recent applications of TMS in REM sleep behavior disorder have unveiled pertinent findings that are predictive of neurodegenerative diseases (8). Furthermore, studies demonstrating the safety and feasibility of rTMS in conditions such as chronic insomnia, obstructive sleep apnea syndrome, restless leg syndrome, and cognitive impairments associated with sleep deprivation have validated its therapeutic efficacy, significantly enriching the primary research scope of rTMS in treating sleep disorders (14–16).

Scientometrics is an interdisciplinary domain that applies mathematical and statistical methods to quantitatively unpack all modalities of knowledge (17). Through scientometric analysis, we can obtain the hot spots, frontiers, and study content in the field. Moreover, the software involved includes CiteSpace (18), VOSviewer (19), HistCite (20), etc. In the previous bibliometric studies of insomnia, there was not only the analysis of insomnia itself (21, 22) but also the bibliometric analysis of insomnia treatment [including cognitive behavioral therapy (23) and aromatherapy (24)], as well as the bibliometric analysis of Chinese herbal compound and acupuncture in the therapeutic method of insomnia, but there was no bibliometric analysis of TMS in the treatment of insomnia. Based on this, we executed this study. The aim of our research is to acquire pivotal information on the hot spots and research content within the field of transcranial magnetic therapy for insomnia through bibliometric analysis, thereby facilitating subsequent researchers in precisely pinpointing information within this domain. The significance of this study lies in its ability to depict the knowledge map, identify the knowledge base and evolution path, and reveal research hot spots and frontier trends through analysis. This provides direct clues for scholars to choose innovative research directions.

## 2 Materials and methods

### 2.1 Data sources

Web of Science primarily emphasizes SCI journals, whereas Scopus boasts a broader scope, being the largest abstract and citation database globally. Scopus encompasses abstracts, references, and indexes of the most extensive scientific and medical literature worldwide. PubMed, on the other hand, centers on biomedical research. In this study, the Web of Science Core Collection, Scopus, and PubMed databases were searched on the same day to mitigate bias stemming from daily database updates. This approach aims to guarantee that the data sources for bibliometric research are comprehensive, authoritative, and varied, ultimately enhancing the accuracy and reliability of the analysis results.

### 2.2 Time span

The time frame for searching the database spans from 1 January 1980 to 31 December 2024.

### 2.3 Inclusion criteria

We have incorporated research articles and comprehensive reviews pertaining to TMS for the treatment of insomnia.

### 2.4 Exclusion criteria

This research has excluded publications that consist of non-original articles.

### 2.5 Search strategy

First of all, we acquired the synonyms for “Transcranial Magnetic Stimulation” and “Insomnia” through the MeSH database in PubMed (25, 26) and then amalgamated the eventual record. Next, we input the WoS with English Topic = “insomnia,” chose “AND” as the logical relation word, and searched the documents. The retrieve strategy is as follows: TS = “insomnia” AND “Transcranial Magnetic Stimulation”; TS = “insomnia” AND “magnetic field therapy”; TS = “insomnia” AND “Magnetic Stimulation, Transcranial”; TS = “Magnetic Stimulations, Transcranial”; TS = “insomnia” AND “noninvasive brain stimulation”; TS = “insomnia” AND “Stimulation, Transcranial Magnetic”; TS = “insomnia” AND “Stimulations, Transcranial Magnetic”; TS = “insomnia” AND “Transcranial Magnetic Stimulation, Paired Pulse”; TS = “insomnia” AND “Transcranial Magnetic Stimulation, Repetitive”; TS = “insomnia” AND “Transcranial Magnetic Stimulation, Single Pulse”; TS = “insomnia” AND “Transcranial Magnetic Stimulations”; TS = “insomnia” AND “TMS”; TS = “insomnia” AND “TBS,” separately. We will rely on the topic words “disorders of initiating and maintaining sleep,” “insomnia disorder,” “primary insomnia,” “secondary insomnia,” “sleep initiation dysfunction,” “sleep initiation, maintenance disorders,” “sleeplessness,” and “transient insomnia” with synonyms for “Transcranial Magnetic Stimulation” to set up the above tactics and then retrieve records from the WoS database. Subsequently, we will set the database search arguments by selecting “Science Citation Index Expand” and “Social Sciences Citation Index” for the “Citation Index” option.

The Scopus database conducts searches within the scope of “TITLE-ABS-KEY,” whereas the PubMed database performs searches using “MeSH Terms.”

### 2.6 Screening process

**Figure 1:**
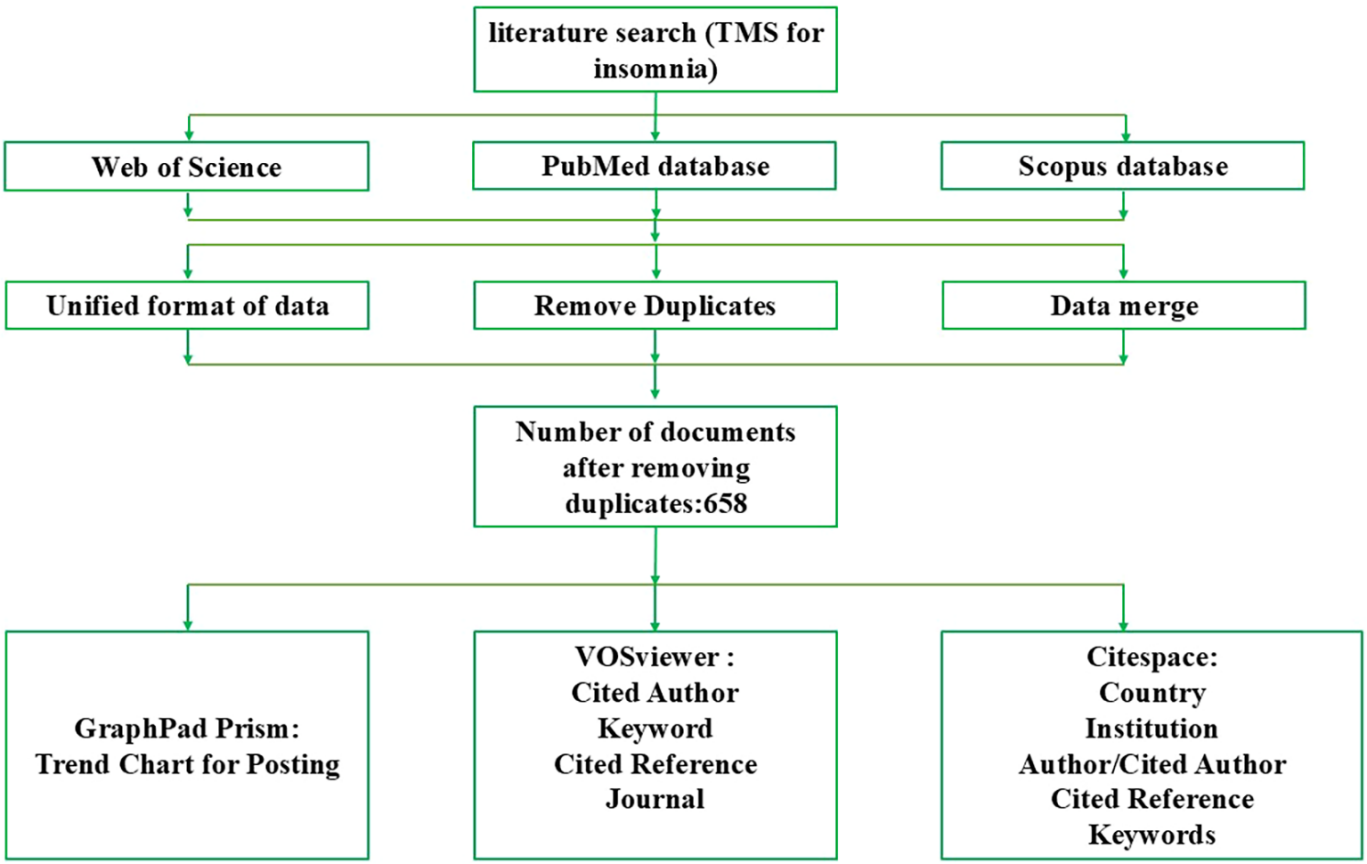
Flowchart of bibliometric analysis on TMS for insomnia.

The screening process for literature is conducted separately according to different databases. Initially, Scopus data are saved in CSV format, while PubMed data are preserved in PMID format. Subsequently, the 2XML tool is utilized to export the PubMed data in XML format. Following this, the data conversion function of the CiteSpace software is employed to convert the data from both the Scopus and PubMed databases into the WoS format. Next, the initial duplicate removal is performed on the data from all three databases using the duplicate removal function in the CiteSpace software, resulting in 50 remaining entries for PubMed, 178 for Web of Science, and 529 for Scopus. Afterwards, the data from these three databases are merged. Finally, a “two-person comparison with a third-party verification” approach is adopted to conduct a second round of duplicate removal on the merged database to ensure data uniqueness, leaving a total of 658 unique records. The flowchart of the bibliometric analysis process is illustrated in **Figure 1**. For specific reference, please adhere to the preliminary guidelines for reporting biomedical bibliometric reviews outlined in the BIBLIO analysis report guidelines (27), as detailed in **Supplementary Table S1**.

### 2.7 Statistics analysis

The retrieved data were imported into CiteSpace 5.1.R8.SE and VOSviewer 1.6.19 software, respectively. Subsequently, leveraging two bibliometric analysis tools, we separately constructed knowledge graphs for countries, institutions, authors, cited authors, keywords, journals, and cited references. Additionally, GraphPad Prism facilitates the creation of trend graphs for scientific research outcomes. Furthermore, CiteSpace provides the module value and silhouette value to appraise the effect of the atlas described, and Q >0.3 indicates that the obtained community structure is significant. When S >0.7, it indicates that the clustering result is the most reliable; if S >0.5, clustering is usually reliable (28). Centrality is an important indicator, and the higher the centrality, the higher the correlation between the keyword and other keywords, belonging to the core of the domain (29).

## 3 Results

### 3.1 Analysis of the scientific research output

**Figure 2:**
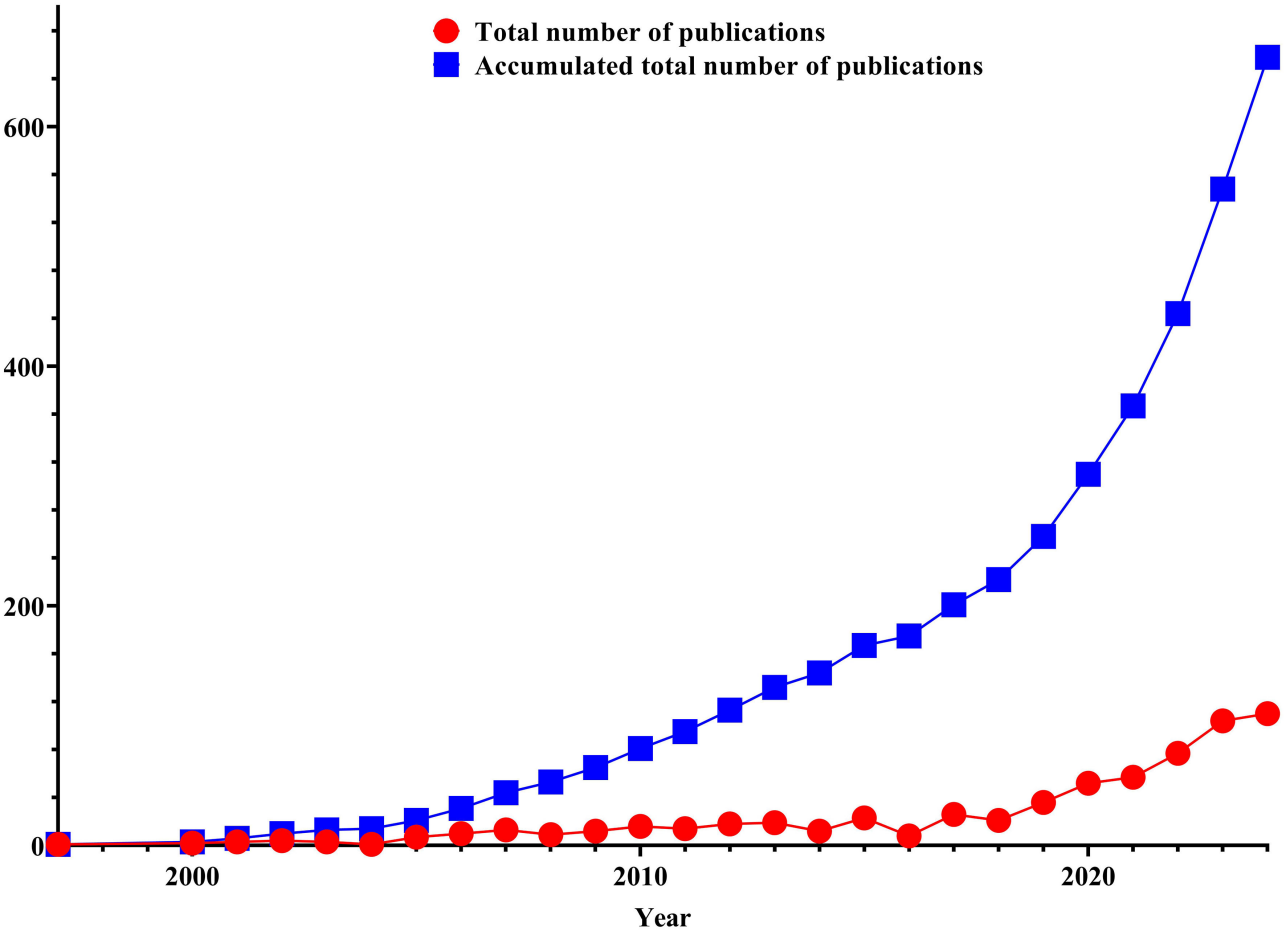
The number of publications and cumulative publication number on TMS for insomnia.

The earliest study can be traced back to 1997. Initially, one article was published, reaching 110 in 2024. As shown in **Figure 2**, from 2000 to 2016, the production of publications remains largely stable at a consistent level, as seen from the median total number of publications, which fluctuates marginally by approximately 9.72. From 2016 to 2024, the total number of publications displayed a conspicuous increasing trend yearly, with an average production steady at approximately 60.375, revealing a key growth drift compared to 1997 to 2016. The above pattern can also be further verified from the accumulated total number of publications, and the holistic drift of the cumulative total number of publications is a rising trend year by year.

### 3.2 Analysis of countries and institutions

**Figure 3:**
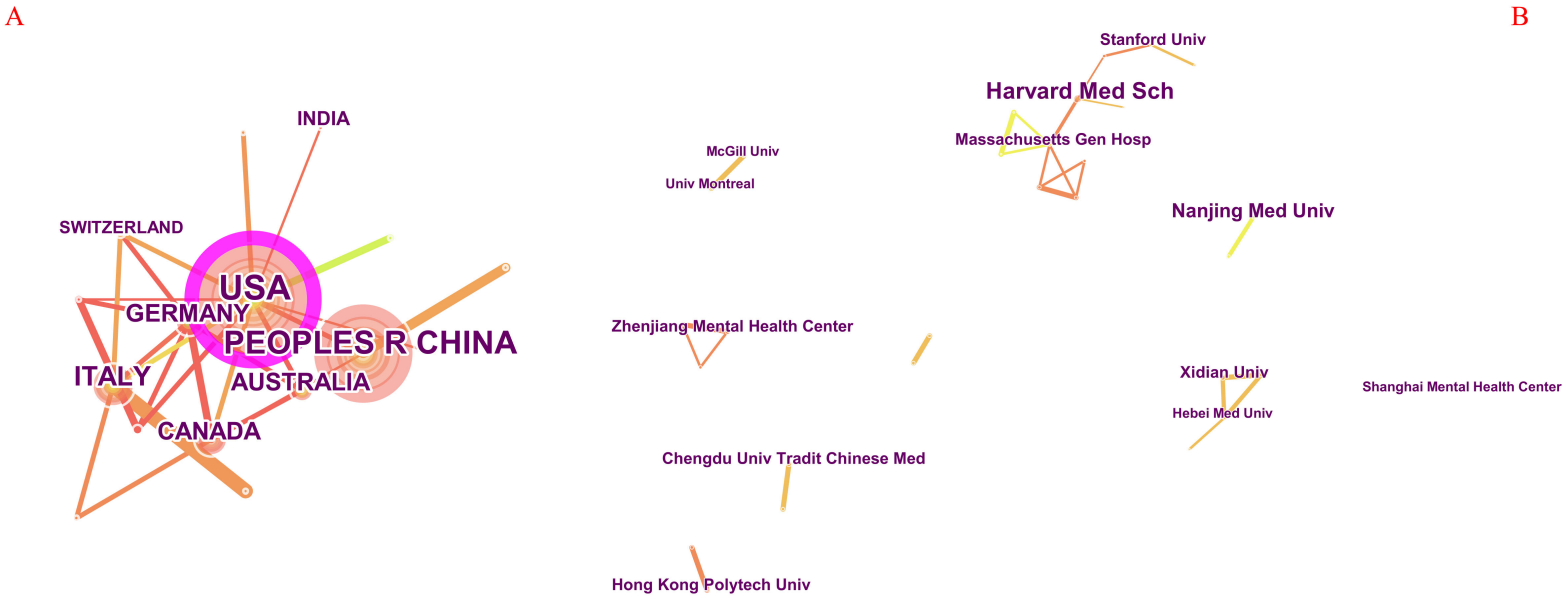
Knowledge map of countries and institutions on TMS for insomnia. **(A)** The author collaboration network, with node sizes signifying the research productivity of respective countries in the field of transcranial magnetic therapy for insomnia. The color purple denotes the significance of nodes, while the lines connecting countries signify their collaborative ties, with thicker lines indicating stronger collaborative relationships. **(B)** The knowledge map of institutional collaboration, where node sizes indicate the relevant scientific research productivity of the institutions, and the lines linking them represent their collaborative ties, with thicker lines signifying closer collaboration.

The study cognate to this relates to 57 countries and 322 organizations, among which the top 5 countries with output are the USA, China, Italy, Germany, and the UK, and the most important countries are the USA (centrality = 0.74) (**Table 1**) and Japan (centrality = 0.48). As revealed in **Figure 3A**, the United States engages in the most collaborations with other nations, encompassing both developed and developing countries, whereas cooperation among the remaining countries is relatively sparse.

**Table 1:** Countries and organizations that contributed to publications on transcranial magnetic stimulation for insomnia.

Country	Documents	Centrality	Organization	Documents	Citations
USA	202	0.74	Harvard Med Sch	9	0.02
China	176	0.20	Nanjing Med Univ	6	0.00
Italy	32	0.07	Stanford Univ	4	0.00
Germany	29	0.07	Xidian Univ	4	0.00
United Kingdom	28	0.07	Massachusetts Gen Hosp	4	0.01
Canada	25	0.00	Chengdu Univ Tradit Chinese Med	4	0.00
Australia	25	0.00	Hong Kong Polytech Univ	4	0.00
Netherlands	12	0.00	Zhenjiang Mental Health Center	4	0.00
India	12	0.07	Univ Montreal	3	0.00
Japan	11	0.48	Hebei Med Univ	3	0.00
Switzerland	11	0.47	McGill Univ	3	0.00
France	10	0.00	Shanghai Mental Health Center	2	0.00
Poland	10	0.00	Mayo Clin	2	0.00
South Korea	8	0.00	Univ Minnesota	2	0.00
Spain	7	0.00	Beijing Normal Univ	2	0.00

In this table, the other two identical columns represent different rankings. The left column of the table represents the countries that have published transcranial magnetic stimulation for insomnia. The frequency of contribution is sorted from high to low.

Harvard Med Sch, Harvard Medical School; Nanjing Med Univ, Nanjing Medical University; Univ Minnesota, University of Minnesota; Beijing Normal Univ, Beijing Normal University; Hebei Med Univ, Hebei Medical University; Massachusetts Gen Hosp, Massachusetts General Hospital; McGill Univ, McGill University; Stanford Univ, Stanford University; Xidian Univ, Xidian University; Chengdu Univ Tradit Chinese Med, Chengdu University of Traditional Chinese Medicine; Hong Kong Polytech Univ, Hong Kong Polytechnic University; Univ Montreal, University of Montreal; Mayo Clin, Mayo Clinic.

The top 3 institutions, arrayed by production, are Harvard Medical School, Nanjing Medical University, and Stanford University, which also have the most cited production. In addition to validating the aforesaid rankings, **Figure 3B** also indicates that the collaborative bearings of the institutions chiefly cover seven sections: a collaborative network centered around Harvard Medical School, Xidian University, Nanjing Medical University, Chengdu University of Traditional Chinese Medicine, Zhejiang Mental Health Center, Hong Kong Polytechnic University, and McGill University as the core nodes. The animated nexus networks of these vast organizations all stem from the USA and China. Moreover, these collaborative bearings are only confined to indigenous higher education institutions and lack collaboration with other national institutions.

### 3.3 Analysis of authors

**Figure 4:**
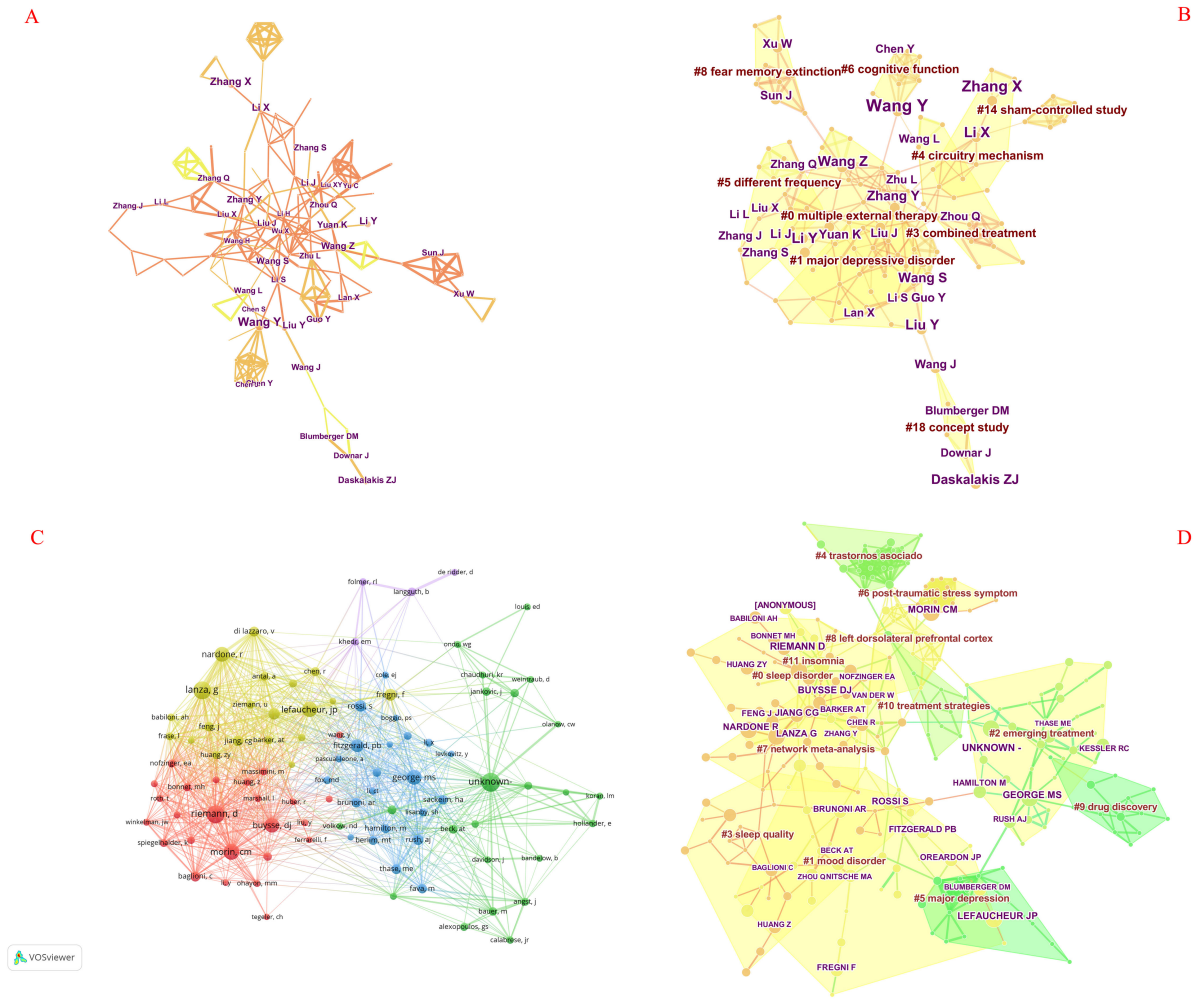
A knowledge graph of authors, cited author, and their collaborative relationships. **(A)** The author collaboration network, with node sizes reflecting the authors’ respective scientific research outputs. The lines connecting the authors signify their collaborative relationships, and thicker lines indicate stronger collaborations. **(B)** The clustering analysis results of the authors, where distinct color blocks represent different clustering categories. The larger the area of a color block, the greater the number of authors engaged in the same research area. **(C)** The collaboration network of cited authors, with node sizes indicating the frequency of citations received by each author. The lines linking the cited authors represent their collaborative ties, and thicker lines denote closer collaborations. **(D)** The clustering analysis results of the cited authors, where different color blocks signify various clustering categories. The larger the area of a color block, the larger the number of cited authors engaged in the same research area.

**Table 2** presents the distribution pattern of authors with a higher number of published articles. The top 5 are Wang Y, Zhang X, Li X, Li Y, and Liu Y (**Figure 4A**). Wang Y, having published the most research, is recognized as the most active author in the field.

**Table 2:** Authors, cited authors, keywords, and cited references contributing to publications on transcranial magnetic stimulation for insomnia.

No.	Author	Frequency	Cited author	Citations	Cited reference	Frequency	Cited reference	Centrality	Keyword	Frequency
1	Wang Y	19	Riemann D	77	Jiang CG (2013)	36	Huang ZY (2018)	0.07	Insomnia	510
2	Zhang X	14	Lefaucheur JP	70	Riemann D (2010)	35	Lanza G (2015)	0.06	Transcranial magnetic stimulation	464
3	Li X	12	Buysse DJ	69	Feng J (2019)	23	Lin J (2019)	0.06	Human	420
4	Li Y	12	Morin CM	69	Huang ZY (2018)	22	Jiang CG (2013)	0.05	Depression	259
5	Liu Y	10	George MS	68	Song PH (2019)	19	Lanza G (2018)	0.03	Anxiety	193
6	Wang Z	10	Lanza G	55	Jiang BH (2019)	18	Li MP (2022)	0.03	Randomized controlled trial	154
7	Daskalakis ZJ	9	Nardone R	55	Lanza G (2015)	18	Zhang Z (2022)	0.03	Headache	150
8	Zhang Y	9	Rossi S	52	Sun NY (2021)	18	Feng J (2019)	0.02	Major depression	136
9	Wang S	9	Jiang CG	50	Babiloni AH (2021)	17	Jiang BH (2019)	0.02	Treatment outcome	119
10	Yuan K	8	Fitzgerald PB	44	Zhang YP (2018)	17	Babiloni AH (2021)	0.02	Controlled study	116

In this table, the other two identical columns represent different rankings. The left column of the table represents the authors who have published transcranial magnetic stimulation for insomnia. The frequency of contribution is sorted from high to low.

By rerunning the CiteSpace software, selecting the pathfinder, pruning the merged network and sliced networks, choosing 4 years per slice in clipping mode, and utilizing the cosine algorithm to obtain clustering analysis results (**Figure 4B**, **Table 3**), nine clustering terms were obtained as follows: #0 multiple external therapy, #1 major depressive disorder, #3 combined treatment, #4 circuitry mechanism, #5 different frequency, #6 cognitive function, #8 fear memory extinction, #18 concept study, and #14 sham-controlled study.

**Table 3:** Authors engaged in transcranial magnetic stimulation for insomnia, detailing knowledge clusters.

Cluster ID	Size	Silhouette	Mean (year)	Label (LLR)	Label (MI)
0	31	0.802	2022	Multiple external therapy	Cue-induced craving (2.44)
1	25	0.748	2022	Major depressive disorder	Cue-induced craving (1.03)
3	14	0.929	2022	Combined treatment	Cue-induced craving (0.32)
4	13	0.904	2022	Circuitry mechanism	Cue-induced craving (0.09)
5	12	0.922	2021	Different frequency	Cue-induced craving (0.2)
6	9	0.991	2020	Cognitive function	Cue-induced craving (0.33)
8	9	0.992	2023	Fear memory extinction	Cue-induced craving (0.23)
18	6	0.934	2017	Concept study	Insomnia disorder (0.07)
14	6	0.992	2020	Sham-controlled study	Insomnia disorder (0.05)

The cluster analysis results mainly include cluster ID, mean year, size, silhouette, label (LLR), and label (MI). Cluster ID is the number after clustering, and Size represents the number of members contained in the cluster. The larger the size, the smaller the number. Mean year represents the average year of the literature in the cluster, which can be used to judge the distance of the cited literature in the cluster. The larger the log-likelihood ratio (LLR), the more representative the cluster category; mutual information (MI) is mainly used to represent the relationship between terms and categories in text mining, and it does not consider the frequency of feature words.

### 3.4 Analysis of cited authors

The analysis of the cited author examines the collective citation of two authors in other scholarly works. When CiteSpace software calculates co-citations of authors, it only considers the co-citations of the first author, and authors who are cited multiple times in the same document are also counted as a single citation (30). By calculating co-citation author relationships, we can obtain an author co-citation network graph, which can indicate the academic community in a certain research field. As shown in **Table 2**, among all co-cited authors, five authors have received over 55 citations each. The authors with the highest number of citations are ranked as Riemann D, Lefaucheur JP, Buysse DJ, Morin CM, and George MS. Additionally, **Figure 4C** depicts the co-citation network graph, which was created after filtering for 22,872 authors who each received over three co-citation counts. We also examined the active collaborations among different co-cited authors; for instance, Riemann D collaborated with Lanza G, Morin CM, and Buysse DJ. Upon rerunning the software, we obtained the clustering analysis results depicted in **Figure 4D**, **Table 4**, identifying a total of 12 distinct clusters, as detailed below: #0 sleep disorder, #1 mood disorder, #2 emerging treatment, #3 sleep quality, #4 trastornos asociado, #5 major depression, #6 post-traumatic stress symptom, #7 network meta-analysis, #8 left dorsolateral prefrontal cortex, #9 drug discovery, #10 treatment strategies, and #11 insomnia.

**Table 4:** Cited authors engaged in transcranial magnetic stimulation for insomnia, detailing knowledge clusters.

Cluster ID	Size	Silhouette	Mean (year)	Label (LLR)	Label (MI)
0	39	0.895	2016	Sleep disorder	Non-sedative hypnotic treatment for insomnia (1.01)
1	35	0.839	2015	Mood disorder	Non-sedative hypnotic treatment for insomnia (0.92)
2	35	0.918	2004	Emerging treatment	Non-sedative hypnotic treatment for insomnia (0.23)
3	24	0.908	2023	Sleep quality	Non-sedative hypnotic treatment for insomnia (1.58)
4	23	0.971	2008	trastornos asociado	Non-sedative hypnotic treatment for insomnia (0.19)
5	19	0.99	2006	Major depression	Non-sedative hypnotic treatment for insomnia (0.14)
6	16	0.959	2017	Post-traumatic stress symptom	Non-sedative hypnotic treatment for insomnia (0.22)
7	15	0.959	2023	Network meta-analysis	Non-sedative hypnotic treatment for insomnia (1.02)
8	14	0.898	2016	Left dorsolateral prefrontal cortex	Systematic review (0.09)
9	14	0.96	2006	Drug discovery	Systematic review (0.09)
10	10	0.957	2009	Treatment strategies	Systematic review (0.1)
11	9	0.97	2011	Insomnia	Systematic review (0.08)

The cluster analysis results mainly include cluster ID, mean year, size, silhouette, label (LLR), and label (MI). Cluster ID is the number after clustering, and Size represents the number of members contained in the cluster. The larger the size, the smaller the number. Mean year represents the average year of the literature in the cluster, which can be used to judge the distance of the cited literature in the cluster. The larger the log-likelihood ratio (LLR), the more representative the cluster category; mutual information (MI) is mainly used to represent the relationship between terms and categories in text mining, and it does not consider the frequency of feature words.

### 3.5 Analysis of journals

**Figure 5:**
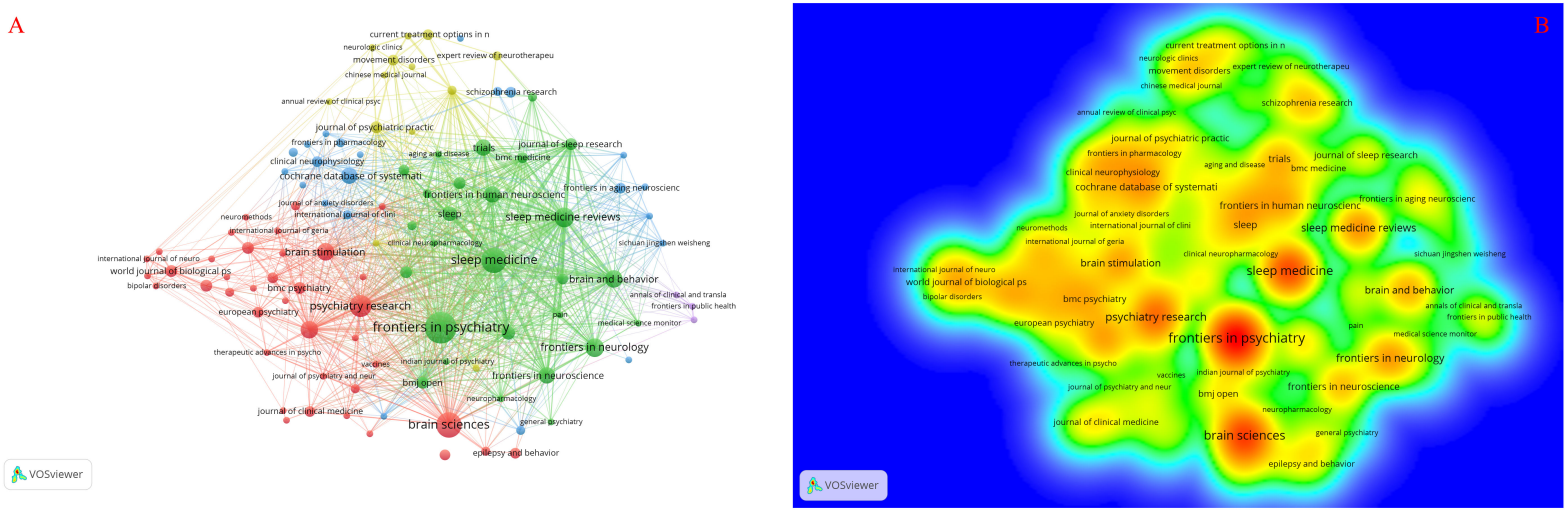
Knowledge graph analysis of journals. **(A)** The journal distribution network, where the size of nodes signifies the volume of related scientific research outputs published by journals. The links between journals depict their collaborative relationships, with thicker lines indicating stronger collaborations. **(B)** The results of the journal cloud-rain chart, where a darker shade of yellow indicates a higher frequency of occurrence, suggesting greater significance of the journal.

The distribution of the fundamental journals undertaken in transcranial magnetic stimulation for insomnia is revealed in **Table 5**. The most prolific journal (**Figures 5A, B**) was Frontiers in Psychiatry (frequency = 24), followed by Brain Sciences (frequency = 17), Sleep Medicine (frequency = 17), Psychiatry Research (frequency = 13), and Sleep Medicine Reviews (frequency = 11); the most critical journal was Neuroimage, followed by Journal of Affective Disorders (centrality = 0.07), American Journal of Psychiatry (centrality = 0.06), Neurology (centrality = 0.06), and Psychiatry Research-Neuroimaging (centrality = 0.06). The median impact factor of the top 10 journals in the field is 5.17, and half of the journals have an IF >3. Therefore, they are cited in the most considerable journals in the Journal co-citation network relationship.

**Table 5:** Journals that contributed to publications on transcranial magnetic stimulation for insomnia.

Journal	Frequency	IF (2023)	Journal	Centrality	IF (2023)
Frontiers in Psychiatry	24	3.2 (Q2)	Neuroimage	0.09	4.7 (Q1)
Brain Sciences	17	2.7 (Q3)	Journal of Affective Disorders	0.07	4.9 (Q1)
Sleep Medicine	17	3.8 (Q1)	American Journal of Psychiatry	0.06	15.1 (Q1)
Psychiatry Research	13	4.2 (Q1)	Neurology	0.06	8.4 (Q1)
Sleep Medicine Reviews	11	11.2 (Q1)	Psychiatry Research-Neuroimaging	0.06	2.1 (Q3)
Frontiers in Neurology	10	2.7 (Q2)	Sleep Medicine	0.05	3.8 (Q1)
Brain and Behavior	9	2.6 (Q2)	JAMA-Journal of the American Medical Association	0.05	63.5 (Q1)
Brain Stimulation	9	7.6 (Q1)	Psychiatry Research	0.04	4.2 (Q1)
Journal of Affective Disorders	9	4.9 (Q1)	Journal of Clinical Psychiatry	0.04	4.5 (Q1)
Cochrane Database of Systematic Reviews	8	8.8 (Q1)	Nature	0.04	50.5 (Q1)

In this table, the other two identical columns represent different rankings. The left column of the table represents the journals that have published transcranial magnetic stimulation for insomnia. The frequency of contribution is sorted from high to low.

### 3.6 Analysis of keywords

**Figure 6:**
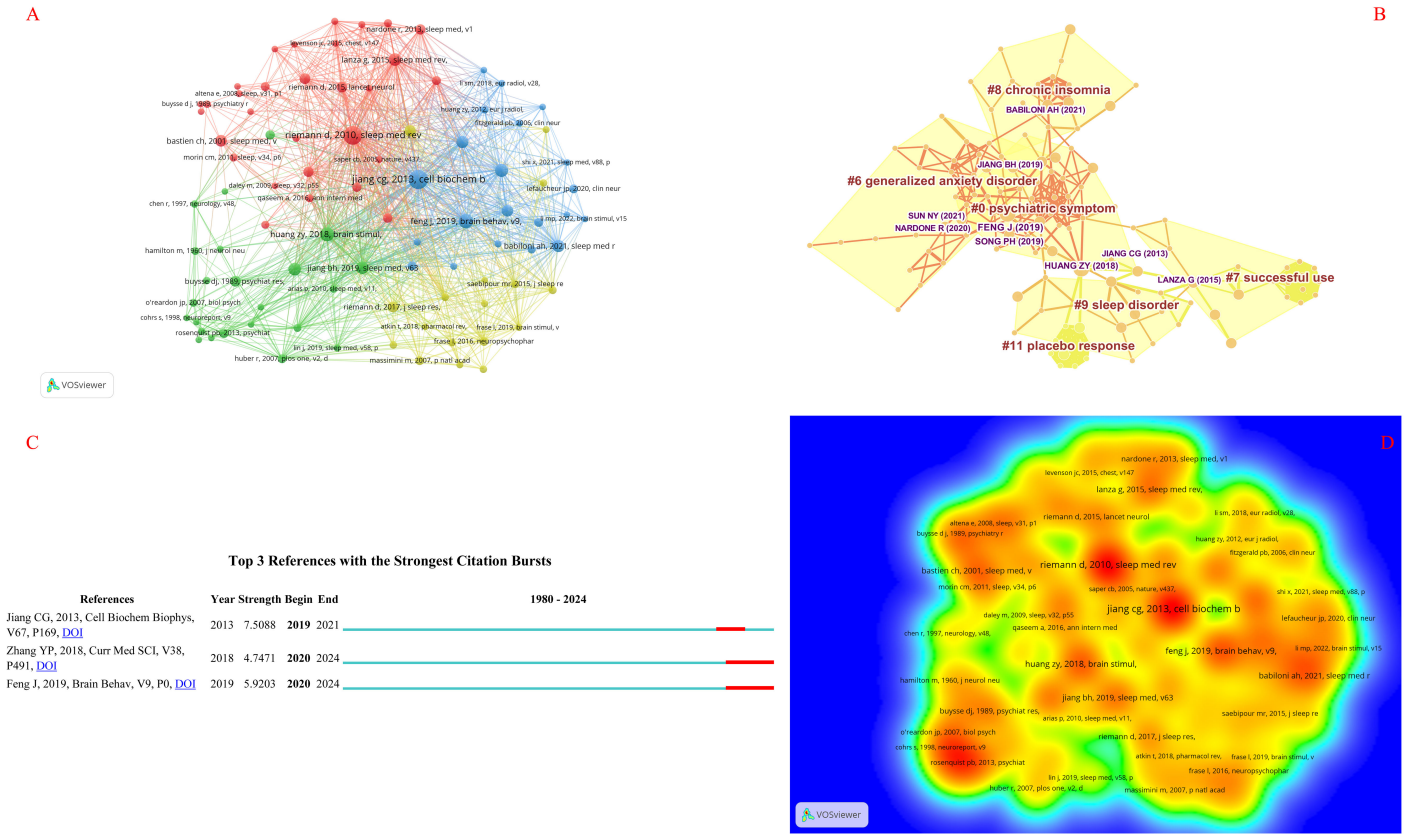
Knowledge graph analysis of keywords. **(A)** A keyword distribution network, in which the size of nodes signifies the frequency of keyword occurrences, and the interconnecting lines represent the relationships between keywords, with thicker lines indicating a stronger correlation between them. **(B)** The results of the keyword clustering density map analysis, where distinct color patches represent different categories. The larger the area of a color patch, the greater the number of researchers engaged in the same field of study. Specifically, green patches represent RCT studies focusing on transcranial magnetic therapy for insomnia, blue patches represent relevant systematic reviews in this domain, and red patches represent studies belonging to other related categories within this field. **(C)** The hot spot distribution network of keywords, with red bars signifying frequently occurring or persistently present keywords, and light green bars indicating keywords that are infrequently cited or have a short duration of relevance. **(D)** The outcomes of the keyword clustering analysis, where different color patches denote distinct clustering categories. The larger the area of a color patch, the more keywords are concentrated within the same research area.

Through co-occurrence analysis of keywords, hot topics in the field of transcranial magnetic stimulation for insomnia can be identified. The frequency of the top 10 keywords in the field outpaced 110, representing the cardinal situation of transcranial magnetic stimulation for insomnia (**Table 2**, **Figure 6A**). Setting aside the two study themes of transcranial magnetic stimulation and insomnia, we found that the study in the field is more focused on the randomized double-blind trials of insomnia combined with depression, headache, and anxiety (**Figure 6B**). As illustrated in **Figure 6C**, the focal point of this field is electroconvulsive therapy, and it also encompasses pharmacological treatments for insomnia accompanied by anxiety and depression.

Subsequently, nine clustering analysis results were obtained, with modularity *Q* = 0.6539 and a mean silhouette of 0.2946, which is greater than 0.5. **Table 6** displays the 10 largest clusters. **Figure 6D** illustrates the co-occurring author keywords and keywords plus, comprising #0, #3, #5, and #6 literature review; #1 high-frequency neuronavigated rtm; #2 cognitive enhancing effect; #4 and #7 Tourette syndrome; #8 new therapy; and #9 related disorder.

**Table 6:** Keywords related to transcranial magnetic stimulation for insomnia, detailing knowledge clusters.

Cluster ID	Size	Silhouette	Mean (year)	Label (LLR)	Label (MI)
0	23	0.716	2005	Literature review	Practical guide (0.91)
1	19	0.968	2005	High-frequency neuronavigated rtm	Extrapyramidal hyperkinetic movement disorder (1.97)
2	17	0.993	2009	Cognitive enhancing effect	Extrapyramidal hyperkinetic movement disorder (4.62)
3	14	0.866	2002	Literature review	Methamphetamine addiction (0.03)
4	12	0.95	2007	Tourette syndrome	Methamphetamine addiction (0.03)
5	9	0.99	2004	Literature review	Methamphetamine addiction (0.03)
6	6	0.976	2002	Literature review	Methamphetamine addiction (0.05)
7	5	0.918	2005	Tourette syndrome	Extrapyramidal hyperkinetic movement disorder (0.03)
8	4	0.95	2005	New therapy	Methamphetamine addiction (0.04)
9	3	0.983	2003	Related disorder	Methamphetamine addiction (0.04)

The cluster analysis results mainly include cluster ID, mean year, size, silhouette, label (LLR), and label (MI). Cluster ID is the number after clustering, and Size represents the number of members contained in the cluster. The larger the size, the smaller the number. Mean year represents the average year of the literature in the cluster, which can be used to judge the distance of the cited literature in the cluster. The larger the log-likelihood ratio (LLR), the more representative the cluster category; mutual information (MI) is mainly used to represent the relationship between terms and categories in text mining, and it does not consider the frequency of feature words.

### 3.7 Analysis of cited reference

**Figure 7:**
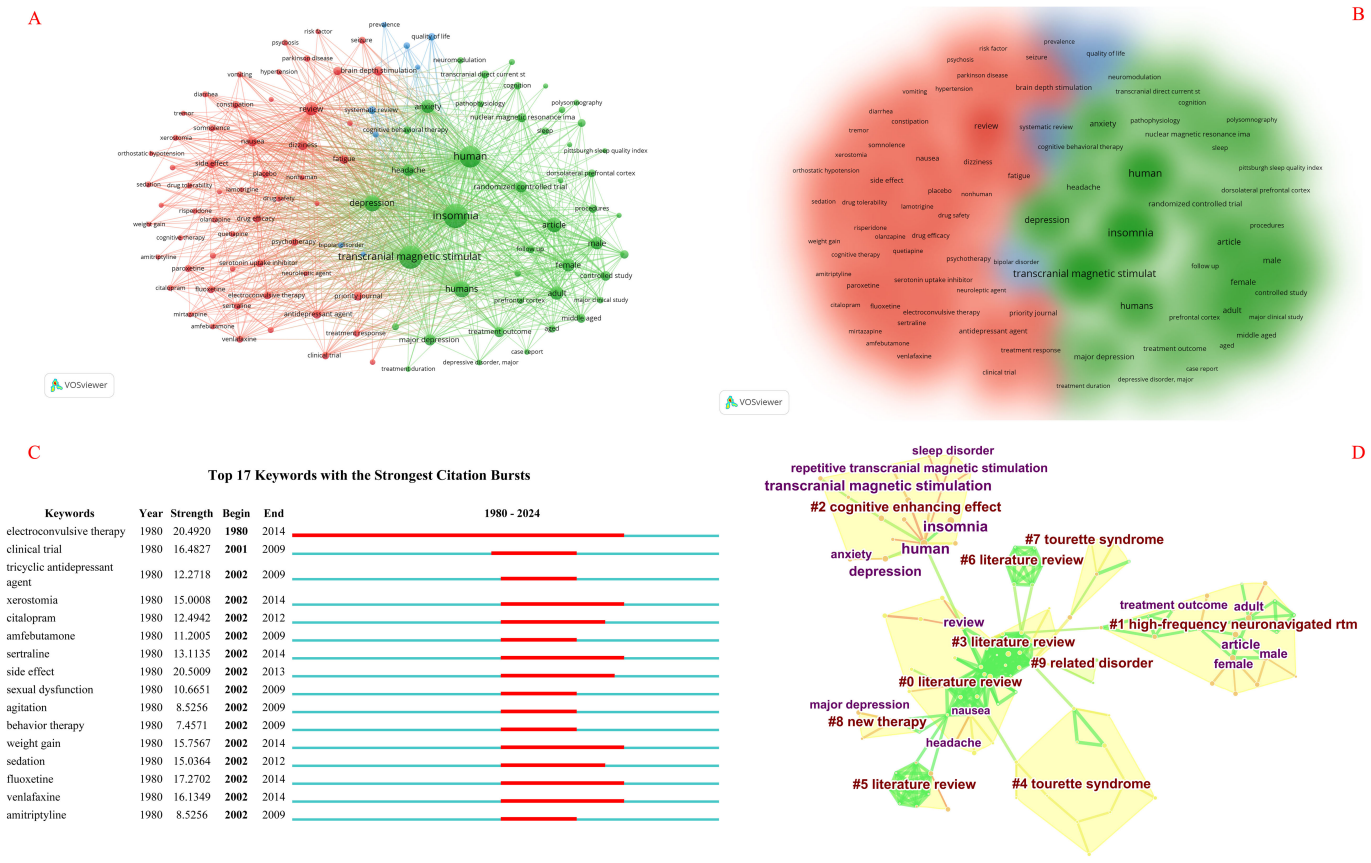
Knowledge graph analysis of cited references. **(A)** The distribution network of cited references, with node size indicating the frequency of citations each reference has received. The lines interconnecting them signify the relationships among the references, and a thicker line indicates a stronger correlation between the two. **(B)** The results of clustering analysis for references, where distinct color blocks represent different clustering categories. A larger color block signifies a greater number of references engaged in the same research area. **(C)** The hot spot distribution network of cited references, where red bars signify frequently cited or persistently relevant references, whereas light green bars indicate those that are infrequently cited or have a shorter duration of relevance. **(D)** The cloud-rain diagram results for the cited references, with a darker yellow hue signifying a higher citation frequency, thereby highlighting the significance of the reference.

Cited references denote instances where two references are cited by a third one. Through the analysis of clustering categories and key nodes within the co-citation network, the knowledge structure of a specific field can be unveiled. **Table 2** displays the top 10 references ranked by frequency and centrality. Specifically, **Figures 7A, D** present the network diagram and cloud-rain diagram of the cited references, where the reference with the highest number of co-citations is Jiang CG (2013) (31) (frequency = 36), followed by Riemann D (2010) (32) (frequency = 35), Feng J (2019) (33) (frequency = 23), Huang ZY (2018) (34) (frequency = 22), and Song PH (2019) (35) (frequency = 19), and the reference with the highest centrality is Huang ZY (2018) (centrality = 0.07) and is deemed the most noteworthy reference in the field, followed by Lanza G (2015) (36) (centrality = 0.06), Lin J (2019) (37) (centrality = 0.06), Jiang CG (2013) (31) (centrality = 0.05), and Lanza G (2018) (38) (centrality = 0.03).

**Figure 7B** presents the co-citation graph of the cited references, unveiling the scientific reciprocity among publications. The network’s modularity, *Q* = 0.8642 >0.7, is deemed reliable. The clustering map, as presented in **Table 7**, highlights the six largest categories, including #0 psychiatric symptom, #6 generalized anxiety disorder, #7 successful use, #8 chronic insomnia, #9 sleep disorder, and #11 placebo response. The clustering results display that the research in the field involves not only insomnia but also includes a systematic review and network meta-analysis of depression and related randomized controlled trials. **Figure 7C** highlights that the hotly cited references in this section include Feng J’s research (33) on the effects of sequential bilateral low-frequency repetitive transcranial magnetic stimulation (rTMS) targeting the dorsolateral prefrontal cortex (DLPFC) on patients with primary insomnia (PI), as well as Zhang YP’s exploration (39) of a randomized trial on the efficacy of acupuncture combined with low-frequency repetitive transcranial magnetic stimulation (rTMS) in treating chronic insomnia.

**Table 7:** Cited references concerned with transcranial magnetic stimulation for insomnia, detailing knowledge clusters.

Cluster ID	Size	Silhouette	Mean (year)	Label (LLR)	Label (MI)
0	37	0.733	2018	Psychiatric symptom	High frequency (1.25)
6	26	0.887	2019	Generalized anxiety disorder	High frequency (0.97)
7	20	0.958	2015	Successful use	High frequency (0.23)
8	18	0.935	2017	Chronic insomnia	Chronic insomnia (0.1)
9	16	0.905	2015	Sleep disorder	Sleep disorder (0.17)
11	10	0.994	2014	Placebo response	Sleep disorder (0.11)

The cluster analysis results mainly include cluster ID, mean year, size, silhouette, label (LLR), and label (MI). Cluster ID is the number after clustering, and Size represents the number of members contained in the cluster. The larger the size, the smaller the number. Mean year represents the average year of the literature in the cluster, which can be used to judge the distance of the cited literature in the cluster. The larger the log-likelihood ratio (LLR), the more representative the cluster category; mutual information (MI) is mainly used to represent the relationship between terms and categories in text mining, and it does not consider the frequency of feature words.

## 4 Discussion

According to the drift of annual publication production, this field has revealed slow development in the past (2000–2016); after 2016, it entered a period of rapid development. This development trend is somewhat related to the history of transcranial magnetic stimulation technology; in 1985, Anthony Barker from the University of Sheffield in the UK truly developed the prototype of a modern transcranial magnetic stimulator, marking the birth of TMS (40). In 1989, Cadwell first developed a repetitive transcranial magnetic stimulation device, laying an important foundation for the application of TMS technology in clinical treatment. In 2008, the US Food and Drug Administration (FDA) approved transcranial magnetic stimulation for the treatment of drug-resistant depression. The article “Evidence-based Clinical Treatment Guidelines for rTMS” was published in the Journal of Clinical Neurology, which clearly states that rTMS can be used to treat psychiatric disorders such as depression, anxiety disorder, obsessive-compulsive disorder, schizophrenia, and substance abuse disorders, as well as pain, movement disorders, stroke, amyotrophic lateral sclerosis, multiple sclerosis, epilepsy, consciousness disorders, Alzheimer’s disease, and neurodegenerative diseases (13). In 2016, the China Food and Drug Administration (CFDA) first approved repeated TMS as an adjunctive treatment for symptoms of depression, anxiety, insomnia, and sexual dysfunction. Based on the above development process, the total number of literature publications and the cumulative total number of publications are closely related to these times.

China and the USA are the chief countries in the study of TMS for insomnia. The countries with the greatest cooperation are between the USA and other countries. Among the top 10 institutions, China and the USA each account for 50%. However, the collaborative relationships among institutions further underscore a pattern, predominantly involving the United States and China as the key cooperating entities. Institutional cooperation is severely lacking. Accordingly, we imply that in-depth cooperation between institutions in various countries can be strengthened to facilitate the rapid progress of TMS for insomnia. International cooperation between institutions is limited, mainly concentrated between the USA and China, with less representation from other geographical regions. A possible solution is to promote transnational and interdisciplinary research networks that can integrate different clinical and cultural perspectives and enrich the overall quality of evidence.

Among journals publishing the most papers, the Sleep Medicine Reviews stands out with an impact factor of 11.2, ranking at the forefront of its field. Frontiers in Psychiatry, with an impact factor of 3.2, is recognized alongside Brain Sciences and Sleep Medicine as the most prolific journals in the field. Neuroimage is the foremost journal in the field. These resources enable us to swiftly identify the key advancements in the field. Moreover, the distribution and centrality of journals indicate that the majority are primarily focused on neuroscience, with fewer being comprehensive in nature. This represents the trajectory for future development within the field.

Through clustering analysis of factors such as the author, cited author, cited reference, and keywords, significant research content regarding the treatment of insomnia with TMS was obtained. Upon integration and summarization, the main aspects involve the following: in terms of types of TMS for insomnia treatment, it encompasses primary insomnia, secondary insomnia, comorbidities of insomnia with other diseases, and related complications; in terms of treatment modalities, in addition to TMS, there are also methods such as massage, acupuncture, and medication. The frequency of choice for rTMS of insomnia is predominantly low frequency at 1 Hz, with stimulation sites including the right posterior parietal cortex and the left/right dorsolateral prefrontal cortex. These represent key findings of this study. The specific details are outlined below: 1) randomized controlled trial (RCT) (sham-controlled study): Research on this topic can be conducted from three perspectives: primary insomnia, secondary insomnia, and the comorbidity of insomnia and anxiety. In terms of treatment methods, there are studies on TMS alone and the combination of acupuncture and TMS for insomnia, both of which constitute important components of research into the treatment of insomnia with TMS. Wei-Chen Lin et al. (41) initiated a study focusing on a randomized controlled trial that utilized low-frequency 1-Hz rTMS to stimulate the left dorsomedial prefrontal cortex for treating insomnia under hypnotic conditions. While both rTMS and sham coils demonstrated comparable therapeutic efficacy for insomnia, this particular rTMS treatment modality stood out in terms of safety, potentially heralding a novel target for future rTMS interventions in insomnia. Qi Zhou et al. (42) conducted a study to validate the effectiveness and safety of treating chronic insomnia patients using transcranial direct current stimulation (tDCS) and rTMS. The results indicated that the combined application of these two interventions could effectively alleviate insomnia symptoms, with notable reductions in HAMD and insomnia factor scores. Furthermore, it exhibited clear advantages in enhancing treatment stability and long-term benefits, offering a safer and more effective alternative for combination therapy in insomnia treatment. Another form of combination therapy involves the use of acupuncture in conjunction with rTMS for the treatment of chronic insomnia. Yangpu Zhang (39) and colleagues treated chronic insomnia by combining acupuncture with low-frequency 1-Hz stimulation at 100% of the resting motor threshold targeting the left prefrontal cortex. Studies have demonstrated that this method not only enhances sleep quality but also improves patients’ quality of life while also offering an additional safe therapeutic option for combination treatments. Notably, this research is highlighted as a key piece of hot spot literature in the cited reference, further confirmed by the hot spot map derived from the citation burst within the cited reference. Zhaoyang Huang et al. (34) examined the efficacy of rTMS in treating the comorbidity of insomnia and generalized anxiety. Their findings revealed that applying rTMS at a frequency of 1 Hz and an intensity corresponding to 90% of the resting motor threshold to the right posterior parietal cortex not only alleviated insomnia but also had a therapeutic impact on anxiety. The therapeutic effect demonstrated a positive correlation with improvements in PSQI and Hamilton Rating Scale for Anxiety (HRSA) scores. This represents an RCT study on the use of transcranial magnetic stimulation for the treatment of comorbid insomnia and anxiety. Regarding secondary insomnia, Jian Jiao and colleagues (43) conducted a randomized controlled trial to investigate the therapeutic efficacy of rTMS in treating insomnia among children with autism. They applied rTMS to the right dorsolateral prefrontal cortex to observe its impact on insomnia as well as other symptoms associated with autism. Furthermore, they analyzed the therapeutic outcomes in conjunction with metabolomics analysis post-treatment. This study not only provides evidence for the use of rTMS in treating insomnia in autism but also elucidates the potential link between sleep disorders and clinical manifestations. After analyzing the content of RCT studies, it was found that rTMS is a non-pharmacological treatment method that is safe and well-tolerated and can effectively improve the subjective sleep quality of insomnia patients. Its main limitations lie in the constraints of small sample size, imperfect blinding, inconsistent parameters, and lack of long-term follow-up. In terms of consistency level, it is highly consistent in safety and subjective improvement, basically consistent in objective sleep parameter improvement, but there is significant controversy in optimal treatment regimen and long-term efficacy. Future research directions should focus on conducting large-sample, multicenter, long-term follow-up RCTs, using more advanced sham stimulation techniques and neuroimaging monitoring, aiming to explore individualized treatment parameters and clarify its effectiveness for different insomnia subgroups, thereby truly promoting TMS to become a clinically standardized treatment for insomnia.

2) Different frequencies: Previous randomized controlled trials have primarily investigated the more pronounced therapeutic effects of low-frequency TMS on insomnia. However, there have been limited studies on the impact of high-frequency TMS on insomnia. This section delves into the selection of frequency and treatment site for TMS in treating insomnia through pertinent research, offering a basis for parameter selection for future researchers to conduct related studies. Xu Xiafei et al. (44) investigated the efficacy of different frequencies of rTMS in treating post-stroke depression (PSD) with insomnia, specifically using low frequency (1 Hz, 90% intensity) targeting the right dorsolateral prefrontal cortex and high frequency (10 Hz, 90% intensity). The findings revealed that the combination of low-frequency rTMS and medication exhibited a notable effect in alleviating insomnia associated with PSD, whereas high-frequency rTMS demonstrated a significant impact on alleviating depressive symptoms in PSD. Furthermore, both frequencies of rTMS showed improvements in neurological deficits and daily living activities. A study conducted by A Irem Sonmez (45) examines the use of high-frequency repetitive transcranial magnetic stimulation (10 Hz rTMS targeting the left dorsolateral prefrontal cortex) to address excessive sleep in depressed adolescents. The study reveals that rTMS can alleviate drowsiness in adolescents with severe depression, yet it falls short in improving insomnia. Moving forward, it is imperative to undertake studies with larger sample sizes, random assignment, and sham controls and incorporate objective quantitative assessments of sleep architecture to comprehensively evaluate the impact of rTMS on sleep in depressed adolescents. Zheng Hui et al. (46) investigated the effects of TMS on insomnia by examining the amplitude of low-frequency fluctuations (ALFF) in resting-state functional magnetic resonance imaging. They utilized a frequency of 1 Hz and applied stimulation to the right dorsolateral prefrontal cortex at an intensity corresponding to 90% of the motor threshold. The findings revealed abnormal spontaneous brain activity within the default mode network (DMN) and frontal-parietal network (FPN) regions among insomnia patients. Furthermore, sleep disturbances were predominantly predictable through functional connectivity within the FPN. Lastly, the study highlighted the potential of this method to specifically target the frontal lobe in middle-aged and elderly individuals. Xumeng Zhao et al. (47) conducted an observation on the power recovery of sleep electroencephalogram (EEG) following low-frequency 1-Hz rTMS stimulation of the left dorsolateral prefrontal cortex for the treatment of insomnia disorder. The results indicated a notable correlation between cortical overactivation and EEG relative power during sleep, as well as sleep measurements in patients with insomnia. This further implies that such treatment may partially ameliorate the relative power abnormalities observed in patients with insomnia disorder. Wu Hongwei et al. (48) investigated the efficacy of rTMS in treating refractory insomnia across various treatment courses and examined the variations in the persistence of clinical outcomes post-treatment cessation. The chosen intervention involved low-frequency 1-Hz stimulation of the right dorsolateral prefrontal cortex. The findings revealed that the duration of efficacy for this approach was associated with the treatment regimen. Notably, even after two consecutive treatment courses spanning 3 months, a certain level of effectiveness persisted, meriting widespread promotion. Song Penghui et al. (35) employed low-frequency 1-Hz rTMS targeted at the right posterior parietal cortex to treat primary insomnia, analyzing the outcomes through EEG network assessments. Their findings revealed a positive treatment effect, with durability spanning at least 1 month, and the ability to revert abnormal alterations in the time-varying EEG network. The aforementioned research underscores that, while the majority of studies on TMS for insomnia opt for low-frequency 1-Hz stimulation, the targeted site varies according to the insomnia subtype. These studies offer valuable insights for clinicians when determining the optimal stimulation site for treating diverse insomnia types. High-frequency rTMS (such as 10 Hz): It typically exerts an excitatory effect on the cortex. Studying its application in insomnia can verify the hypothesis that “excitatory control of brain regions involved in the initiation and maintenance of sleep (such as the pathway from the hypothalamic preoptic area to the arousal center), or enhancement of sleep homeostasis pressure.” A more direct theory is that high-frequency stimulation of the DLPFC may reduce overall excessive arousal by re-establishing its “top-down” inhibitory control over the limbic system (such as the amygdala). Low-frequency rTMS (such as 1 Hz): It typically exerts an inhibitory effect on the cortex. The significance of studying it lies in verifying the hypothesis: “promoting sleep by inhibiting the overly active arousal center (such as the DLPFC itself).” This is more directly targeted at the “high arousal” model of insomnia. Main controversies and future directions: The debate over the “optimal frequency” is still inconclusive. Is low frequency better, or is high frequency better? Or does it vary from person to person? Larger-scale clinical studies are needed to verify this. Target-frequency interaction: The optimal frequency may vary when stimulating different targets. For example, 10 Hz is used to excite the left DLPFC, while 1 Hz is used to inhibit the right DLPFC. This different frequency stimulation pattern between the left and right hemispheres (similar to the treatment regimen for depression) has not been extensively studied in insomnia. Individualized predictive biomarkers: How to find the optimal frequency for each patient in practical clinical settings with low cost and high efficiency is a top priority for future research. The effectiveness of low-frequency (typically 1 Hz) rTMS stimulation of the DLPFC lies in its precise targeting of the core pathophysiological mechanisms of chronic insomnia—cognitive hyperarousal and emotional regulation dysfunction. This represents a “treatment to the cause” approach, rather than simple sedation. Zhu Lin et al.’s research (16) primarily focuses on the functional characteristics of low-frequency DLPFC-rTMS treatment in patients with chronic insomnia, introducing an additional method for assessing and predicting rTMS responsiveness. It provides recent high-quality evidence for the treatment of chronic insomnia disorders with low-frequency rTMS (right DLPFC). Ma Haixia et al.’s systematic review and meta-analysis (9) synthesized multiple studies, demonstrating that rTMS can effectively improve PSQI and expand our understanding of rTMS in improving objective sleep parameters, including SE, SOL, TST, WASO, and NA. These studies confirm its effectiveness. In summary, the superiority of the low-frequency rTMS stimulation of the DLPFC protocol lies in its precise neural regulation based on modern understanding of the neural mechanisms underlying insomnia. Relevant literature provides strong support from multiple perspectives, including clinical efficacy (RCT), network mechanisms (fMRI), and theoretical models.

3) Literature review: This section employs systematic review and meta-analysis methods to assess various aspects, including the efficacy of rTMS in treating insomnia, the effectiveness and placebo response of multimodal therapy for primary insomnia, the efficacy and safety of diverse external treatments for insomnia patients, and the application of rTMS in polysomnography for examining the electrophysiology of primary insomnia. The insights gained from randomized controlled trials in this field offer pertinent evidence-based guidance for clinicians. Binghu Jiang’s research (49) has revealed that the placebo effect is notably and consistently significant in alleviating insomnia symptoms, with 65.9% of the efficacy of multimodal treatment actually attributable to the placebo condition. Zhen Wang (50) compared the efficacy and safety of multiple external therapies for insomnia patients, discovering that both massage and rTMS exhibit significant effects in enhancing sleep quality. However, in terms of safety, rTMS falls short. Consequently, massage can be considered the primary choice for improving sleep quality. These constitute vital components of new therapeutic approaches for insomnia. Yongliang Zheng’s research (51) indicates that rTMS could be an effective technology for enhancing the sleep architecture of primary insomnia. However, to avoid considering studies influenced by the “first-night effect” of PSG, it may be sensible to opt for a shorter duration of treatment sessions, extending the overall treatment period beyond 2 weeks and incorporating the use of rTMS in conjunction with medication. Additionally, given the heterogeneity, the typical effects remain undefined. Exploring a more viable method to furnish more objective, evidence-based support would hold greater significance for further research endeavors. Nianyi Sun et al. (52) have discovered that rTMS can enhance sleep quality by augmenting slow-wave and rapid eye movement (REM) sleep, suggesting its potential as a safe and effective treatment for insomnia. Nevertheless, further international, multicenter, high-quality RCTs, coupled with more objective assessments and follow-up evaluations tied to quality of life, are required. This common conclusion has emerged from systematic reviews and meta-analyses of rTMS treatment for insomnia to date. 4) Generalized anxiety and severe depression: Tang Sijie and colleagues (53), through retrospective analysis, discovered that personalized rTMS treatment for severe depression and generalized anxiety disorder is safe and potentially offers sustained enhancements in sleep, quality of life, and emotional symptoms for individuals with affective disorders. Yang Shuai’s research (54) integrates functional near-infrared spectroscopy and polysomnography to demonstrate an elevated activation level in the prefrontal cortex of MDD patients with insomnia, which correlates with both subjective and objective sleep quality measures. This correlation exhibits an inverse relationship with total sleep time and sleep efficiency, while positively correlating with PSQI scores. Furthermore, this study serves to affirm the prevalent research focus in this field, which primarily revolves around electroconvulsive therapy (ECT) and treatments for anxiety and depression. ECT is typically employed when pharmacological interventions are ineffective or not tolerable for patients. TMS, a non-invasive brain stimulation technique, stimulates the cerebral cortex via magnetic fields to enhance brain function. It is frequently utilized in the management of mild to moderate depression, anxiety, and other psychiatric disorders. 5) Cognitive function: The research focus of Yuan Jie and his colleagues (55) is on the influence of magnetic stimulation at the Shenmen acupoint on cognitive function in individuals with chronic insomnia. The aim of this study is to assess the cognitive function, sleep quality, and emotions of patients with chronic insomnia; observe the clinical effectiveness of magnetic stimulation at the Shenmen acupoint in addressing cognitive impairments in these patients; and analyze the potential mechanisms involved. This research offers a novel therapeutic perspective for treating insomnia using rTMS via acupuncture at specific acupoints. 6) Fear memories disappear: Sun Jingjing et al. (56) primarily investigated the effectiveness of rTMS in treating insomnia with regard to the eradication of fear memory. The aim of this preliminary study is to confirm whether 1-Hz rTMS stimulation can enhance fear extinction memory among patients with insomnia disorders and to ascertain whether sleep alterations mediate this impact. 7) Sleep disorder: Jiang Yilin et al. (57) explored the impact of rTMS on comorbid sleep disorders in preschool children with attention deficit hyperactivity disorder (ADHD). The integration of rTMS with parental behavior management training appears to be more efficacious, not only mitigating sleep disorder symptoms in ADHD children but also extending the longevity of treatment effectiveness. Eman M. Khedr’s research (58) assessed the effectiveness of rTMS in addressing sleep disorders associated with Parkinson’s disease. It involved stimulating the right and left parietal regions at a frequency of 20 Hz and administering 10 sessions of 20 Hz rTMS across both parietal cortices. This approach led to an improvement in sleep quality among Parkinson’s patients, as evidenced by both subjective and objective measurements. Furthermore, the therapeutic outcomes were observed to be in tandem with enhancements in motor and emotional functionalities. 8) Psychiatric symptom: Charlotte E. Luff et al. (59) discovered through a literature review that neural modulation has the capacity to regulate neuronal activity underlying sleep. Nevertheless, studies leveraging neural modulation to enhance objective measurements of sleep are scarce. While the majority of findings regarding the improvement in subjective measurements of sleep quality are reproducible, they are plagued by significant methodological constraints and a potent placebo effect.

In summary, all the aforementioned research contents constitute vital components within this field. The primary effective frequency of treatment lies in the low-frequency range of 1 Hz, with the dorsolateral prefrontal cortex being the preferred site for stimulation. While the number of related RCT studies is gradually increasing, they do possess certain limitations. The scarcity of randomized controlled trials and the methodological quality of the research may represent the primary constraints of these studies, which also pose major challenges for future RCT research to address. Additionally, exploring the combined use of other non-invasive adjuvant therapies, such as acupuncture and massage, with TMS for insomnia treatment, while utilizing polysomnography and fMRI to monitor relevant indicators, represents a promising research direction for future endeavors, offering fresh perspectives for clinical research. Furthermore, in the research on transcranial magnetic stimulation for insomnia treatment, the combination of fuzzy similarity method and random forest, as studied by Giuliana Bilotta (60), has provided us with a robust analytical framework: fuzzy similarity is responsible for front-end understanding, which acknowledges and quantifies the ambiguity and continuity in clinical reality, transforming qualitative knowledge of human experts into computable mathematical forms. Random forest is responsible for back-end mining, which robustly searches for predictive patterns and biomarkers in high-dimensional and complex feature spaces.

## 5 Conclusions

This study offers new perspectives on the trends in TMS for treating insomnia. Despite its limitations, it illustrates the global trajectory of TMS in insomnia, displaying it in a knowledge graph for readers. The USA and China lead in the number of research papers published in this field, with cooperation among nations predominantly centered around networks between the USA and other countries. As for institutional collaboration, it is confined to higher education institutions in both China and the USA. Wang Y is the author with the most relevant papers, and Riemann D, the most-cited author, is notable. The journal with the most publications in this field is Frontiers in Psychiatry, and the momentous journal is Neuroimage. The keywords primarily revolve around depression, anxiety, and RCTs, excluding insomnia and TMS; Huang ZY (2018) is noteworthy. Additionally, future research should concentrate on large-scale RCTs and cohort studies to further verify their clinical effectiveness. In conclusion, the findings of this study could offer researchers valuable insights into cutting-edge topics, potential collaborators, countries and institutions of interest, promising research subjects, and current hot spots in the field.

## Data Availability

The original contributions presented in the study are included in the article/**Supplementary Material**. Further inquiries can be directed to the corresponding author.
